# Quantifying the benefits of healthy lifestyle behaviors and emotional expressivity in lowering the risk of COVID-19 infection: a national survey of Chinese population

**DOI:** 10.1186/s12889-023-17158-6

**Published:** 2023-11-30

**Authors:** Yudong Miao, Wanliang Zhang, Yi Li, Jian Wu, Zhanlei Shen, Junwen Bai, Dongfang Zhu, Ruizhe Ren, Jingbao Zhang, Dan Guo, Clifford Silver Tarimo, Chengpeng Li, Wenyong Dong

**Affiliations:** 1 Department of Health Management, College of Public Health, Zhengzhou University, Henan, China; 2 Department of Neurology, Henan Provincial People’s Hospital, People’s Hospital of Zhengzhou University, Henan, China; 3 Department of Science and Laboratory Technology, Dar es salaam Institute of Technology, Dar es Salaam, Tanzania; 4 Department of Human Resources, Henan Provincial People’s Hospital, People’s Hospital of Zhengzhou University, Henan, China; 5 Department of Hypertension, Henan Provincial People’s Hospital, People’s Hospital of Zhengzhou University, Henan, China

**Keywords:** COVID-19 Infection, Lifestyle behavior, Emotional expressivity, China

## Abstract

**Background:**

COVID-19 is still prevalent in most countries around the world at the low level. Residents’ lifestyle behaviors and emotions are critical to prevent COVID-19 and keep healthy, but there is lacking of confirmative evidence on how residents’ lifestyle behaviors and emotional expressivity affected COVID-19 infection.

**Methods:**

Baseline study was conducted in August 2022 and follow-up study was conducted in February 2023. Baseline survey collected information on residents’ basic information, as well as their lifestyle behaviors and emotions. Follow-up study was carried out to gather data on COVID-19 infection condition. Binary logistic regression was utilized to identify factors that may influence COVID-19 infection. Attributable risk (AR) was computed to determine the proportion of unhealthy lifestyle behaviors and emotional factors that could be attributed to COVID-19 infection. Sensitivity analysis was performed to test the robustness of the results.

**Results:**

A total of 5776 participants (46.57% males) were included in this study, yielding an overall COVID-19 infection rate of 54.8% (95%CI: 53.5 – 56.0%). The findings revealed that higher stress levels [aOR = 1.027 (95%CI; 1.005–1.050)] and lower frequency in wearing masks, washing hands, and keeping distance [aOR = 1.615 (95%CI; 1.087–2.401)], were positively associated with an increased likelihood of COVID-19 infection (all *P* < 0.05). If these associations were causal, 8.1% of COVID-19 infection would have been prevented if all participants had normal stress levels [Attributable Risk Percentage: 8.1% (95%CI: 5.9-10.3%)]. A significant interaction effect between stress and the frequency in wearing masks, washing hands, and keeping distance on COVID-19 infection was observed (β = 0.006, *P* < 0.001), which also was independent factor of COVID-19 infection.

**Conclusions:**

The overall COVID-19 infection rate among residents is at a medium level. Residents’ increasing stress and decreasing frequency in wearing masks and washing hands and keeping distance contribute to increasing risk of infection, residents should increase the frequency of mask-wearing, practice hand hygiene, keep safe distance from others, ensure stable emotional state, minimize psychological stress, providing evidence support for future responses to emerging infectious diseases.

**Supplementary Information:**

The online version contains supplementary material available at 10.1186/s12889-023-17158-6.

## Introduction

As of April 2023, available data highlights the ongoing prominence of COVID-19 (Coronavirus disease 2019) as a significant global health concern, with an estimated 764 million individuals affected by the virus worldwide [1]. Despite the significant effort it has taken, COVID-19 (Coronavirus disease 2019) continues to persist at relatively low levels and viral infections remain an unavoidable occurrence [2]. Although the World Health Organization has declared that “COVID-19 is no longer a public health emergency of international concern” [3], many years of ongoing pandemics have had a huge impact on countries around the world. However, it is important to acknowledge that the complete cessation of the COVID-19 virus has not been attained, underscoring the imperative for ongoing and sustained efforts in the long-term prevention and management of the virus among individual residents to reduce the burden of healthcare systems.

Studies have shown that COVID-19 infection is associated with persistent inflammation associated with multiple organs in the human body [4, 5], while inflammatory factors are also associated with other post-infection syndromes, such as post-infectious fatigue syndrome [6, 7], which pose a significant threat to the human body. However, some studies have shown that residents’ healthy lifestyle behaviors including smoking cessation, moderate alcohol consumption, regular exercise, always wearing masks, always washing hands, keeping distance, and healthy psychological emotion, can effectively prevent the inflammation [8–11]. Moreover, a study showed that compared with people with no chronic disease, people with severe chronic disease had higher COVID-19 infection risk, the severe chronic disease also increases the risk of inflammation, then increases the likelihood of COVID-19 infection [12]. And study had shown a gender differences in COVID-19 infection, with men being more susceptible to COVID-19 than women [13].

In addition, it is worth noting that lifestyle and emotional factors exhibit mutual influence, as evidenced by prior research demonstrating a robust association between lifestyle and psychological emotion [14–17]. These interrelated dynamics may have implications for various viral infections including COVID-19 [8, 9]. However, since the outbreak of the COVID-19 pandemic, few studies have been conducted on the quantitative association between lifestyle and psychological emotion and COVID-19 infection condition, and the interaction between lifestyle and emotion on COVID-19 infection condition also has not been established yet.

In response to the global epidemic situation, China has changed the COVID-19 management measures for managing the COVID-19 outbreak [18]. This involved a gradual lifting of large-scale lockdown measures. However, this transition has resulted in a notable increase in the COVID-19 infection rate among residents. Although the lethality of the virus has decreased, this has, to some extent, added complexity to the control of the COVID-19 virus. In addition, it is important to consider the rapid mutation rate of COVID-19 virus strain. In China, the predominant epidemic strains have undergone a shift from BA.5.2 and its subclades, as well as BF.7 and its subclades, to the emergence of the XXB strain [19]. Although some studies have shown that vaccines are an effective means of preventing infectious diseases (including COVID-19 virus), reducing strain infection and improving herd immunity [20–24], the development and research of vaccines has not effectively kept pace with the mutation speed of the COVID-19 virus, and there are now large numbers of people who are re-infected with COVID-19 virus in most areas, which are still the important issues for the prevention and control of the COVID-19. Therefore, it is crucial for residents to prioritize their self-protection measures by adopting specific lifestyle practices and maintaining a favorable emotional state that can play a significant role in effectively preventing the spread of COVID-19 infection.

This study examined the lifestyle behaviors and emotional status of residents prior to COVID-19 infection and quantitatively assessed their impact on the likelihood of infection. The purpose of this study is to analyze the impact of the lifestyle and emotional status of pre-infection residents on COVID-19 infection, provide new ideas for subsequent COVID-19 virus prevention and control and treatment, and reduce the medical cost of the healthcare system. This quantitative study can provide valuable guidance and evidence support for residents to effectively prevent worldwide prevalent unknown emerging infectious diseases (including COVID-19) outbreak in future.

## Methods

### Study setting, participants and procedure

From June 29, 2022 to July 2, 2022, we recruited online volunteers and used a self-designed questionnaire to conduct a preliminary survey. Then we conducted a dynamic study of adults (aged ≥ 18 years old) in four areas of Jiangsu province (eastern region), Henan province (central region), Heilongjiang province (northeast region), and Qinghai province (western region) of China were selected to form a representative sample using stratified random sampling. Subsequently, within each area, more than two rural areas and two urban areas from each city were selected randomly. From August. 3, 2022 to August. 14, 2022, baseline survey was conducted in the aforementioned four regions, followed by a subsequent follow-up with the baseline population in from February. 1, 2023 to February. 18, 2023. This timeline allowed for the collection of comprehensive data to examine the evolution of factors under investigation over time.

**Fig. 1:**
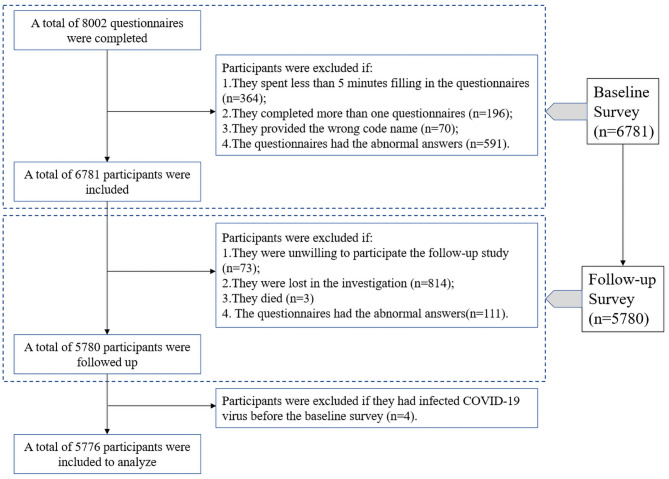
Inclusion and exclusion of participants

During the baseline survey conducted in July 2022, data was collected from 8002 participants. After applying exclusion criteria, information from 6781 participants was ultimately included in the current study. In January 2023, China began to implement the “Class B epidemic and B Management” policy. Then, in February 2023, a follow-up survey was conducted, in which we followed up 5780 participants in the study population based on the baseline survey, with a follow-up rate of 85.24%. We then excluded four adults who had been infected with the COVID-19 virus in the baseline survey, and the remaining 5776 participants were included in our analysis and study (Fig. 1). We make assurance that there have not happened any changes in the violence and type of virus during follow up period. The study was approved by the Life Science Ethics Review Committee of the Life Sciences Ethics Review Committee of Zhengzhou University, and participants signed informed consent forms on the questionnaire.

### Assessment

During the baseline survey, we investigated all valid information about participants before infection, and during the follow-up survey, we continued to investigate the follow-up information of these participants, the questionnaire for some independent variables measurements is found in Additional file 1: Table S1.

### Sociodemographic covariates

The study included 7 covariates: gender (categorized as men or women), age(divided into 5 groups: 18–29, 30–39, 40–49, 50–59, and ≥ 60), region of residence (classified as Jiangsu, Henan, Heilongjiang, or Qinghai province based on population density data from the seventh population census of China, Additional file 1: Fig. S1), marital status (categorized as married or other), education level (categorized into junior and below, senior, undergraduate, graduate and above), presence of chronic disease (yes/no), and history of allergic (categorized as yes, no, or not clear).

### Lifestyle behaviors assessment

The study assessed four potential lifestyle behavior factors based on definitions and criteria derived from the American College of Lifestyle Medicine (ACLM). These factors included smoking (categorized as smoke, quit, or never. We defined the smoking as “refers to personal use of cigarettes or other tobacco products, such as cigars, pipes, waterpipes, etc.”), drinking (categorized as drink, quit, or never. We defined the drinking as “personal consumption of any beverage containing more than 0.5% alcohol, including beer, wine, spirits, etc.”), physical exercise (categorized as never, < 1 time, 1–2 times, 3–5 times, or ≥ 6 times per week. We defined the physical exercise as “a sport, exercise, or athletic activity that requires physical exertion and energy expenditure.”), and adherence to preventive measures such as wearing masks, washing hands, and keeping distance (categorized as never, seldom, sometimes, often, or always. We defined the wearing masks, washing hands, and keeping distance as adherence to preventive measures) (Additional file 1: Table S2).

To effectively reflect the lifestyle of different groups, we calculated the lifestyle behaviors score using the simple way to sum scores, we assigned values to each lifestyle option based on the criteria established for the study [25]. These values ranged from negatively to positively, or from less frequency to higher frequency, reflecting the extent to which each lifestyle choice aligned with the desired criteria. We allocated numeric values to denote the various smoking statuses (smoke = 1 point, quit = 2 points, never = 3 points), scoring ranged 1–3 points. Similarly, drinking was assigned as (drink = 1 point, quit = 2 points, never = 3 points), scoring ranged 1–3 points. Moreover, physical exercise was assigned as (never = 1 point, < 1 time = 2 points, 1–2 times = 3 points, 3–5 times = 4 points, ≥ 6 times = 5 points), scoring ranged 1–5 points. Additionally, wearing masks, washing hands, and keeping distance were scored accordingly (never = 1 point, seldom = 2 points, sometimes = 3 points, often = 4 points, always = 5 points), scoring ranged 1–5 points. With higher scores indicating healthier lifestyle behaviors.

To conduct the attributable risk (AR) analysis, we classified lifestyles as either health or unhealthy based on the mean value of the lifestyle behaviors scores. Based on the mean scores obtained, we categorized the smoking status options. Since the mean score for smoking status was 2.51, we defined a mean score of 2.51 as whether or not a criterion for whether or not a healthy smoking status is, we classified option 3 (never) as healthy lifestyle choice, while option 1 (smoke) and 2 (quit) were classified as unhealthy. Similarly, for drinking status, with a mean score of 2.44, we defined a mean score of 2.44 as whether or not a criterion for whether or not a healthy drinking status is, we categorized option 3 (never) as a health choice, while option 1 (drink) and 2 (quit) were considered unhealthy. In terms of physical exercise, where the mean score was 3.40, we defined a mean score of 3.40 as whether or not a criterion for whether or not healthy physical exercise is, options 4 (3–5 times) and 5 (≥ 6 times) were classified as healthy choices, whereas options 1 (never), 2 (< 1time), and 3 (1–2 times) were classified as unhealthy. Lastly, for wearing masks, washing hands, and keeping distance, the mean score was 4.15. we defined a mean score of 4.15 as whether or not a criterion for whether or not healthy preventive measures are. Here, option 5 (always) was deemed a healthy choice, while options 1 (never), 2 (seldom), 3 (sometimes), and 4 (often) were classified as unhealthy. The AR formula is as following:

$${\rm{AR}}\,{\rm{(Attribute}}\,{\rm{Risk)}}\,{\rm{ = }}\frac{{Ie - Io}}{{Ie}}*100\%$$

Notes: *Ie* indicates the incidence of the exposed group and *Io* indicates the incidence of the non-exposed group.

### Emotional expressivity assessment

We evaluated emotional expressivity through three emotional factors, namely anxiety, depression and stress. The assessment of these scores utilized the internationally validated emotional self-assessed “DASS-21” scale (Depression, Anxiety and Stress Scale, a maturity scale validated in China [26, 27], Additional file 1: Table S3). Anxiety was measured through seven questions, including items: “2. I was aware of dryness of my mouth”, “4. I experienced breathing difficulty (e.g., excessively rapid breathing, breathlessness in the absence of physical exertion)”, “7. I experience trembling (e.g., in the hands)”, “9. I was worried about situations in which I might panic and make a fool of myself)”, “15. I felt I was close to panic”, “19. I was aware of the action of my heart in the absence of physical exertion (eg, sense of heart rate increase, heart missing a beat)”, “20. I felt scared without any good reason”. Depression was assessed using seven questions with items: “3. I couldn’t seem to experience any positive feeling at all”, “5. I found it difficult to work up the initiative to do things”, “10. I felt I had nothing to look forward to”, “13. I felt down-heart and blue”, “16. I was unable to become enthusiastic about anything”, “17. I felt I wasn’t worth much as a person”, “21. I felt that life was meaningless”. Stress level was evaluated through seven questions: “1. I found hard to wind down”, “6. I tend to over-react to situations”, “8. I felt that I was using a lot of nervous energy”, “11. I found myself getting agitated”, “12. I found it difficult to relax”, “14. I was intolerant of anything that kept me from getting on with what I was doing”, “18. I felt that I was rather touchy” [28, 29]. We tested the DASS-21 scale for reliability and validity. The results showed that the Cronbach’s alpha coefficient was 0.955, indicating that the scale reliability was reliable.

For the calculation of the emotional score, we assigned values (no = 0 points, sometimes = 1 point, often = 2 points, always = 3 points) for the 4 options (no, sometimes, often, always) of 21 questions according to the emotional evaluation criteria in DASS-21. According to international standards, the total score for anxiety, depression and stress was multiplied by 2 to get the final score. The total score for anxiety, depression, and stress ranged from 0 to 42, with higher scores indicating higher levels of anxiety, depression, and stress [30].

For the purpose of conducting AR analysis, we established cut-off values for anxiety, depression, and stress based on internationally recognized standards. According to these standards, a score of ≤ 7 in anxiety was categorized as normal, while a score of ≥ 8 was considered abnormal. For depression, a score of ≤ 9 was classified as normal, while a score of ≥ 10 was deemed abnormal. Similarly, in the case of stress, a score of ≤ 14 was considered normal, while a score of ≥ 15 was classified as abnormal [31].

### Interaction of lifestyle behaviors and emotional factors

To capture the combined effects of lifestyle behaviors and emotional factors, we employed multiplication interaction by multiplying the respective factors. In the current study, we investigated four lifestyle behavior factors and three emotional expressivity factors. Based on the correlation observed between these factors, we identified a total of eight interactions. These interactions included the interaction between drinking status and stress, the interaction between physical exercise and anxiety, the interaction between physical exercise and depression, the interaction between physical exercise and stress, the interaction between wearing masks, washing hands, and keeping distance and anxiety, the interaction between wearing masks, washing hands, and keeping distance and depression, and the interaction between wearing masks, washing hands, and keeping distance and stress.

### Dependent variable

We used the question in the questionnaire “Did you get infected with the COVID-19 virus?“ The option 1 (yes, confirmed cases by doctors in medical institutions), option 2 (yes, abnormal nucleic acid testing, antigen testing, virus culture isolation or serological test results), option 3 (presence of clinical symptoms of COVID-19 infection), option 4 (abnormal clinical test results: such as chest X-ray, chest CT test, lung function, blood oxygen saturation, blood routine, etc.) were combined into 1 = had infected COVID-19 virus while the option 5 (no) and option 6 (not clear)were combined into 0 = no. If “had infected COVID-19 virus” was selected, participants were asked which symptoms they had experienced.

### Statistical analysis

We used the chi-square (χ^2^) test to analyze the difference in COVID-19 infection rates of residents with different characteristics, used the mean ± standard deviation to describe the lifestyle behaviors of Chinese residents prior to infection, used the median (upper and lower quartiles) to describe the emotional status of Chinese residents prior to infection. Considering that the infection rate of the population may have regional clustering, we gave priority to using the multi-level statistical model (also known as the random-effects model), and first fit the two-level empty model, taking the region (province) as the high level. The intra-class correlation (ICC) was used to determine whether the variation of the data was clustered in the high level (province). The test result showed that ICC was 0.0322046, indicating that only 3.22046% of the variation of dependent variables was caused by the high level (Province), much less than the general level of 0.1 (10%), indicating that the degree of variation in the dependent variable (infection) is low and the aggregation is low, which may not fit the multilevel model analysis, then we used multi-factor regression analysis. Binary logistic regression was used to explore the influencing factors of COVID-19 infection condition while correlation analysis was used to assess the correlation between lifestyle behavior factors and emotional expressivity factors. We used correlation analysis to analyze the correlation between lifestyle and emotional expressivity, then we used multiplicative interactions to calculate interaction terms, and finally we put the interaction terms as dependent variables into the regression model to explore the magnitude of the interaction effect [32–34]. A collinearity test using the variance inflation factor (VIF) (< 6, less than the cut-off value of VIF “10”) was used to determine the correlation between independent variables. No collinearity was detected between these covariates. To assess the impact of unhealthy lifestyle behavior factors and abnormal emotional factors on COVID-19 infections, we estimated the attributable risk (AR) analysis that enables us to estimate the proportion of infections that could be attributed to these factors. Additionally, sensitivity analysis was performed to ensure the stability and robustness of the results whereas residents with history of allergic were excluded to ensure consistency of the findings. All analyses were performed using SPSS 27 and STATA 17 software, with *P* < 0.05 considered as statistically significant.

## Results

### Baseline characteristics of residents prior to infection and their COVID-19 infection condition

The findings showed that 5776 participants eventually completed the follow-up survey, of which 46.57% were men and 53.43% were women. The overall COVID-19 infection rate was 54.8% (53.5 − 56.0%). Among individuals aged 60 years and older, the infection rate was found to be the lowest, with a rate of 50.3% (47.4 − 53.2%). In terms of regional differences, residents from Jiangsu province had the highest infection rate which stood at 67.1% (65.2 − 69.1%). When considering education status, residents with postgraduate and above, exhibited the highest infection rate, reaching 66.3% (56.3 − 76.3%). In addition, the infection rate of residents with the history of allergic was 65.5% (61.0 − 70.0%), and the infection rate of residents who drank was relatively high [56.4% (53.8 − 58.9%)] (Table 1).

**Table 1:** Baseline characteristics prior to infection and COVID-19infection condition of residents

Variables	Participants (%)	Infection status	
		Infected % (95%CI)^a^	*p* value^b^
**total**	5776 (100)	54.8 (53.5–56.0)	
**Socio-demographic**			
**Gender**			0.955
Man	2690 (46.57)	54.7 (52.8–56.6)	
Woman	3086 (53.43)	54.8 (53.0-56.6)	
**Age**			0.018
18–29	727 (12.59)	55.0 (51.4–58.6)	
30–39	1579 (27.34)	56.6 (54.1–59.0)	
40–49	1119 (19.37)	55.2 (52.3–58.1)	
50–59	1205 (20.86)	56.0 (53.2–58.8)	
≥ 60	1146 (19.84)	50.3 (47.4–53.2)	
**Region**			<0.001
Jiangsu province	2210 (38.26)	67.1 (65.2–69.1)	
Henan province	2419 (41.88)	47.7 (45.8–49.7)	
Heilongjiang province	965 (16.71)	43.9 (40.8–47.1)	
Qinghai province	182 (3.15)	54.9 (47.6–62.2)	
**Marital status**			0.061
Married	5082 (87.98)	48.4 (47.1–49.6)	
Others	694 (12.02)	51.4 (47.7–55.2)	
**Education status**			<0.001
Junior and below	2478 (42.90)	48.2 (46.3–50.2)	
Senior	1416 (24.42)	51.7 (49.1–54.3)	
Undergraduate	1793 (31.04)	65.6 (63.4–67.8)	
Graduate and above	89 (1.54)	66.3 (56.3–76.3)	
**Chronic disease**			0.046
Yes	937 (16.22)	57.7 (54.6–60.9)	
No	4839 (83.78)	54.2 (52.8–55.6)	
**The history of allergic**			<0.001
Yes	429 (7.43)	65.5 (61.0–70.0)	
No	4695 (81.28)	54.0 (52.5–55.4)	
Not clear	652 (11.29)	53.4 (49.5–57.2)	
**Lifestyle behaviors**			
**Smoking status**			0.290
Yes	1241 (21.49)	52.9 (50.2–55.7)	
Quit	345 (5.97)	56.8 (51.6–62.1)	
Never	4190 (72.54)	55.1 (53.6–56.6)	
**Drinking status**			0.350
Yes	1469 (25.43)	56.4 (53.8–58.9)	
Quit	321 (5.56)	53.6 (48.1–59.1)	
Never	3986 (69.01)	54.3 (52.7–55.8)	
**Physical exercise**			0.016
6 times and above	1042 (18.04)	52.9 (49.8–55.9)	
3–5 times	1863 (32.25)	55.1 (52.9–57.4)	
1–2 times	1823 (31.55)	56.1 (53.8–58.3)	
Below 1 time	479 (8.28)	58.7 (54.2–63.1)	
Never	569 (6.38)	49.6 (45.4–53.7)	
**Wearing masks, washing hands, keeping distance**			<0.001
Never	164 (2.84)	45.1 (37.4–52.8)	
Seldom	311 (5.38)	63.3 (58.0-68.7)	
Sometimes	458 (7.93)	60.7 (56.2–65.2)	
Often	2393 (41.43)	55.2 (53.3–57.2)	
Always	2450 (42.42)	52.7 (50.8–54.7)	
**Emotional expressivity**			
**Anxiety**			0.083
Normal (0–7)	5225 (90.46)	54.4 (53.0-55.7)	
Abnormal (≥ 8)	551 (9.54)	58.3 (54.1–62.4)	
**Depression**			0.225
Normal (0–9)	5363 (92.85)	54.4 (53.2–55.9)	
Abnormal (≥ 10)	413 (7.15)	57.6 (52.8–62.4)	
**Stress**			0.191
Normal (0–14)	5584 (96.68)	54.6 (53.3–55.9)	
Abnormal (≥ 15)	192 (3.32)	59.4 (52.4–66.4)	

Note:

^a^ %, percentage; 95%CI, 95% confidence interval;

^b^ Difference between categories within each variable

### Lifestyle behaviors and emotional expressivity prior to Infection of residents

Table 2 presented the mean scores and standard deviations of different lifestyle behavior factors, the distribution of emotions. The total mean score for smoking status was 2.51 ± 0.82, indicating the average level of smoking behavior among the participants. The mean score for drinking was 2.44 ± 0.87, reflecting the average level of alcohol consumption. In terms of physical exercise, the total mean score was 3.34 ± 1.17, representing the average frequency and intensity of physical exercise reported by the participants. Finally, the mean score for wearing masks, washing hands, and keeping distance was 4.15 ± 0.98, indicating the overall adherence to these preventive measures. Among the emotional expressivity assessed, including anxiety, depression, and stress, all participants showed the optimal scores. The findings also reveal that women exhibit healthier behaviors compared to men in terms of smoking, drinking, and adherence to preventive measures such as wearing masks and washing hands. Contrarily, in terms of emotional well-being, women tend to experience higher levels of stress compared to men, with scores of 0 (0, 4) for women and 0 (0, 2) for male participants. Residents with chronic disease were found to be more stressed than their counterparts without chronic diseases [0 (0, 6) VS 0 (0, 2)]. Residents with the history of allergic were more stressed than residents without the history of allergic [2 (0, 10) VS 0 (0, 2)].

**Table 2:** Lifestyle behaviors and emotional expressivity prior to infection of residents

Variables	Lifestyle behaviors				Emotional expressivity		
	Smoking status (M ± SD)^a^	Drinking status (M ± SD)^a^	Physical exercise (M ± SD)^a^	Wearing masks, washing hands, keeping distance (M ± SD)^a^	Anxiety Me (lower Quartile, upper Quartile)^b^	Depression Me (lower Quartile, upper Quartile)^b^	Stress Me (lower Quartile, upper Quartile)^b^
**Total**	2.51 ± 0.82	2.44 ± 0.87	3.40 ± 1.17	4.15 ± 0.98	0 (0, 0)	0 (0, 0)	0 (0, 0)
**Gender**							
Man	1.99 ± 0.94	1.93 ± 0.95	3.40 ± 1.19	4.09 ± 0.99	0 (0, 2)	0 (0, 2)	0 (0, 2)
Woman	2.97 ± 0.25	2.87 ± 0.47	3.40 ± 1.15	4.20 ± 0.96	0 (0, 2)	0 (0, 2)	0 (0, 4)
**Age**							
18–29	2.66 ± 0.74	2.57 ± 0.80	3.31 ± 1.10	4.28 ± 0.95	0 (0, 2)	0 (0, 2)	0 (0, 4)
30–39	2.56 ± 0.81	2.4 ± 0.88	3.22 ± 1.13	4.28 ± 0.96	0 (0, 2)	0 (0, 2)	0 (0, 4)
40–49	2.44 ± 0.87	2.37 ± 0.90	3.46 ± 1.15	4.21 ± 0.94	0 (0, 2)	0 (0, 2)	0 (0, 4)
50–59	2.44 ± 0.86	2.39 ± 0.90	3.53 ± 1.17	4.09 ± 0.95	0 (0, 2)	0 (0, 0)	0 (0, 2)
≥ 60	2.50 ± 0.80	2.45 ± 0.84	3.54 ± 1.22	3.90 ± 1.02	0 (0, 2)	0 (0, 0)	0 (0, 2)
**Region**							
Jiangsu province	2.53 ± 0.81	2.49 ± 0.84	3.40 ± 1.13	3.89 ± 1.05	0 (0, 4)	0 (0, 2)	0 (0, 4)
Henan province	2.54 ± 0.81	2.44 ± 0.87	3.32 ± 1.19	4.26 ± 0.91	0 (0, 2)	0 (0, 0)	0 (0, 2)
Heilongjiang province	2.46 ± 0.87	2.34 ± 0.92	3.60 ± 1.15	4.45 ± 0.84	0 (0, 2)	0 (0, 2)	0 (0, 4)
Qinghai province	2.23 ± 0.95	2.21 ± 0.93	3.63 ± 1.17	4.30 ± 0.89	0 (0, 4)	0 (0, 6)	0 (0, 8)
**Marital status**							
Married	2.49 ± 0.83	2.42 ± 0.88	3.41 ± 1.16	4.14 ± 0.97	0 (0, 2)	0 (0, 2)	0 (0, 2)
Others	2.66 ± 0.74	2.57 ± 0.81	3.37 ± 1.18	4.21 ± 0.99	0 (0, 2)	0 (0, 2)	0 (0, 4)
**Education status**							
Junior and below	2.47 ± 0.85	2.44 ± 0.87	3.44 ± 1.23	4.00 ± 1.02	0 (0, 2)	0 (0, 0)	0 (0, 2)
Senior	2.41 ± 0.87	2.37 ± 0.89	3.42 ± 1.16	4.23 ± 0.92	0 (0, 2)	0 (0, 2)	0 (0, 2)
Undergraduate	2.63 ± 0.75	2.49 ± 0.85	3.35 ± 1.08	4.30 ± 0.92	0 (0, 4)	0 (0, 2)	0 (0, 6)
Graduate and above	2.81 ± 0.58	2.47 ± 0.85	3.30 ± 1.13	4.24 ± 0.89	0 (0, 6)	0 (0, 4)	0 (0, 6)
**Chronic disease**							
Yes	2.39 ± 0.85	2.35 ± 0.88	3.45 ± 1.20	3.86 ± 1.02	0 (0, 4)	0 (0, 2)	0 (0, 6)
No	2.53 ± 0.82	2.45 ± 0.87	3.39 ± 1.16	4.21 ± 0.96	0 (0, 2)	0 (0, 2)	0 (0, 2)
**The history of allergic**							
Yes	2.58 ± 0.78	2.45 ± 0.86	3.28 ± 1.12	4.21 ± 0.94	2 (0, 6)	0 (0, 2)	2 (0, 10)
No	2.50 ± 0.83	2.43 ± 0.87	3.46 ± 1.15	4.16 ± 0.97	0 (0, 2)	0 (0, 0)	0 (0, 2)
Not clear	2.55 ± 0.80	2.46 ± 0.85	3.10 ± 1.25	4.09 ± 1.05	0 (0, 4)	0 (0, 4)	0 (0, 6)

Note:

^a^ M ± SD, mean ± standard deviation;

^b^ Me (lower Quartile, upper Quartile). Me, Median; lower Quartile, 25th quartile; higher Quartile, 75th quartile

### Influencing factors of the COVID-19 infection condition among the residents

**Fig. 2:**
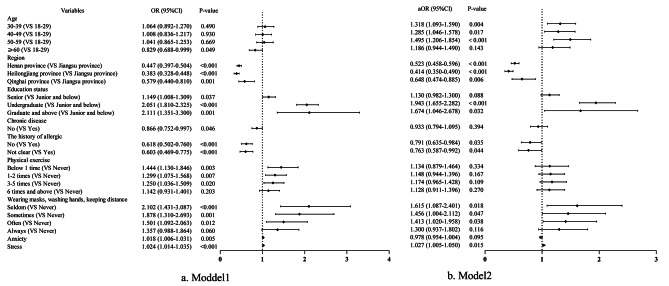
Influencing factors of COVID-19 infection condition of residents Note: Model1, unadjusted; Model2, adjusted for significantly statistical variables, including age, region, education status, chronic disease, the history of allergic, physical exercise, wearing masks, washing hands, keeping distance, anxiety, stress. OR, odds ratio; aOR, adjusted odds ratio; 95%CI, 95% confidence interval

After adjusting for the potential confounding factors (age, region, education status, chronic disease, the history of allergic, physical exercise, wearing masks and washing hands and keeping distance, anxiety, and stress), younger age was associated with the higher likelihood of COVID-19 infection [OR = 1.318 (95%CI; 1.093–1.590), *P* = 0.004; OR = 1.285 (95%CI; 1.046–1.578), *P* = 0.017; OR = 1.495 (95%CI; 1.206–1.854), *P* < 0.001]. The odds ratio of contracting COVID-19 was significantly lower in other provinces than Jiangsu province [OR = 0.523 (95%CI; 0.458–0.596), *P* < 0.001; OR = 0.414 (95%CI; 0.350–0.490), *P*<0.001; OR = 0.648 (95%CI; 0.474–0.885), *P* = 0.006]. Additionally, individuals with higher levels of education had a higher risk of COVID-19 infection compared to those with lower levels of education [OR = 1.943 (95%CI; 1.655–2.282), *P* < 0.001; OR = 1.674 (95%CI; 1.046–2.678), *P* = 0.032]. Furthermore, individuals without a history of allergic had a lower risk of COVID-19 infection compared to those with a history of allergic [OR = 0.791 (95%CI; 0.635–0.984), *P* = 0.035]. The lower the frequency of wearing masks and washing hands and keeping distance, the greater the likelihood of COVID-19 infection [OR = 1.615 (95%CI; 1.087–2.401), *P* = 0.018; OR = 1.456 (95%CI; 1.004–2.112), *P* = 0.047; OR = 1.413 (95%CI; 1.020–1.958), *P* = 0.038]; In addition, the higher the stress of the population, the higher the likelihood of COVID-19 infection [OR = 1.027 (95%CI; 1.005–1.050), *P* = 0.015] (Fig. 2 and Additional file 1: Table S4).

### The effect of interactions between lifestyle behaviors and emotional expressivity prior to infection on the COVID-19 infection condition

We first analyzed the correlation between lifestyle behavior factors and emotional factors using correlation analysis (Additional file 1: Table S5). Subsequently, the impact of lifestyle behavior and emotional factors on the status of COVID-19 infection was assessed, revealing a noteworthy association between stress and drinking status, which exhibited a significant positive effect on the COVID-19 infection status (*β* = 0.008). Specifically, higher levels of stress were found to correspond to more severe drinking habits, thereby increasing the likelihood of contracting COVID-19. The study also revealed that there was a significant positive association between COVID-19 infection status and the interaction between anxiety and physical exercise (*β* = 0.004). In other words, higher anxiety levels were associated with lower frequency of physical exercise, which in turn was linked with a higher likelihood of contracting COVID-19. The interaction of stress and physical exercise had a significant effect on infection status (*β* = 0.006), that is the higher the stress, the lower the frequency of physical exercise, the higher the likelihood of COVID-19 infection. The interaction between anxiety and wearing masks and washing hands and keeping distance had a promoting effect on COVID-19 infection condition (*β* = 0.004), that is the more anxious, the lower the frequency of wearing masks and washing hands and keeping distance, the higher the COVID-19 infection likelihood. The study findings indicated a significant positive association between the interaction of stress and the frequency of wearing masks, washing hands, and keeping distance with COVID-19 infection condition (β = 0.006). This suggests that higher stress levels were associated with a lower frequency of adhering to protective measures such as wearing masks, washing hands, and keeping distance, consequently increasing the likelihood of COVID-19 infection (Table 3).

**Table 3:** Effect of interactions between lifestyle behaviors and emotional expressivity on COVID-19 infection condition among residents

Variables		Emotional expressivity		
		Anxiety	Depression	Stress
Lifestyle behaviors	Smoking status	~	~	~
	Drinking status	~	0.004^a^	0.008^a^ ***
	Physical exercise	0.004^a^ *	0.002^a^	0.006^a^ ***
	Wearing masks, washing hands, keeping distance	0.004^a^ *	0.003^a^	0.006^a^ ***

Note:

~, There are no data;

^a^ the *β* value;

*p < 0.05, **p < 0.01, ***p < 0.001.

### Symptoms of infection in infected people in different lifestyle behaviors and emotional expressivity

**Fig. 3:**
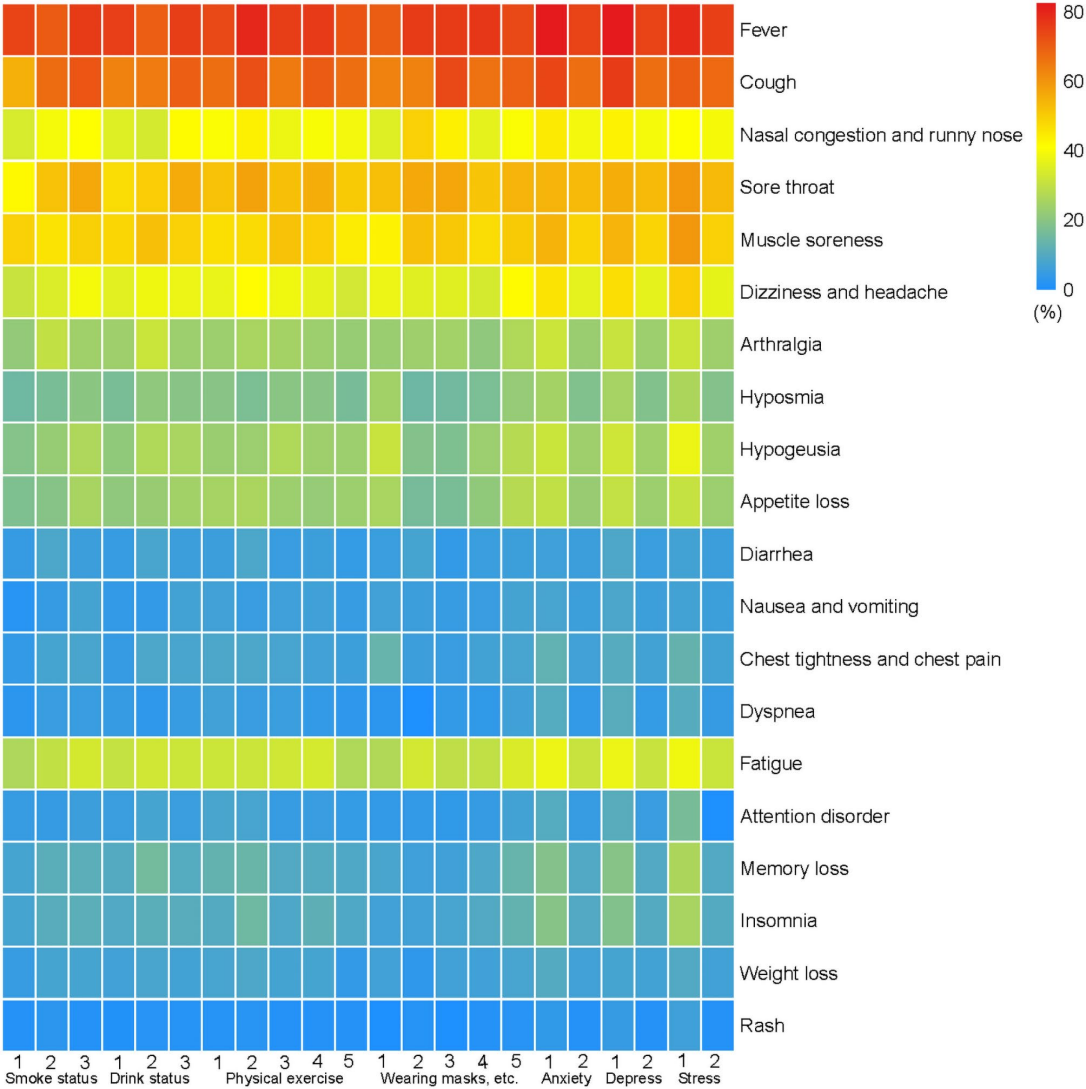
Symptoms of infection in different lifestyle behaviors and emotional expressivity of infected people Note: Smoke: 1 = yes, 2 = quit,3 = no; Drink: 1 = yes, 2 = quit,3 = no; Physical exercise: 1 = never, 2 = below 1 time, 3 = 1–2 times, 4 = 3–5 times, 5 = 6 times and above; Wearing masks, washing hands, keeping distance: 1 = never, 2 = seldom, 3 = sometimes, 4 = often, 5 = always; Anxiety: 1 = abnormal, 2 = normal; Depression: 1 = abnormal, 2 = normal; Stress: 1 = abnormal, 2 = normal

The findings suggested that fever, cough, nasal congestion, sore throat, muscle soreness, dizziness, headache and fatigue were the main symptoms of COVID-19 infection in all infected people. (Fig. 3 and Additional file 1: Table S6).

### Attributable risk analysis and sensitivity analysis

According to Table 4, the analysis revealed that among all infected individuals, 2.9% of the infections were attributed to drinking and quitting (indicating alcohol consumption), 1.6% were associated with engaging in physical exercise less than or equal to twice a week, and 6.4% were linked to a lower frequency of wearing masks, washing hands, and keeping distance. Additionally, 8.1% of the infections were attributed to abnormal stress, 6.7% to abnormal anxiety, and 5.6% to abnormal depression. Finally, we excluded people with a history of allergies and then conducted the sensitivity analysis, we found no significant change in the influencing factors affecting the COVID-19 infection condition. Education status, wearing masks and washing hands and keeping distance in lifestyle behaviors, and stress in emotional factors remained important independent factors in COVID-19 infection condition (Additional file 1: Table S7).

**Table 4:** Attributable risk analysis of COVID-19 infection condition in the lifestyle behaviors and emotional expressivity of residents

Variables	Difference between incidence in the exposed group and incidence in the non-exposed group	Attributable risk percentage (ARP) % (95%CI)^a^
**Smoking status**		
Healthy (3)		
Unhealthy (1, 2)	-1.3	-2.4% (-1.1% - -3.7%)
**Drinking status**		
Healthy (3)		
Unhealthy (1, 2)	1.6%	2.9% (1.5-4.2%)
**Physical exercise**		
Healthy (4, 5)		
Unhealthy (1, 2, 3)	0.9%	1.6% (0.6-2.7%)
**Wearing masks, washing hands, keeping distance**		
Healthy (5)		
Unhealthy (1, 2, 3, 4)	3.6%	6.4% (4.4-8.4%)
**Anxiety**		
Normal (0–7)		
Abnormal (≥ 8)	3.9%	6.7% (4.7-8.7%)
**Depression**		
Normal (0–9)		
Abnormal (≥ 10)	3.2%	5.6% (3.7-7.4%)
**Stress**		
Normal (0–14)		
Abnormal (≥ 15)	4.8%	8.1% (5.9-10.3%)

Note:

Smoke: 1 = yes, 2 = quit,3 = no;

Drink: 1 = yes, 2 = quit,3 = no;

Physical exercise: 1 = never, 2 = below 1 time, 3 = 1–2 times, 4 = 3–5 times, 5 = 6 times and above;

Wearing masks, washing hands, keeping distance: 1 = never, 2 = seldom, 3 = sometimes, 4 = often, 5 = always

^a^ 95%CI, 95%confidence interval

## Discussion

In the study, which began in August 2022 and ended in February 2023, during which the domestic COVID-19 prevention and control policy changed. We found that residents had a COVID-19 infection rate of 54.8%, residents had kept healthy lifestyle behaviors and emotions prior to infection. And we also found that pre-infection lifestyle behaviors and emotional expressivity were associated with COVID-19 infection condition: decreasing frequency in wearing masks, washing hands and keeping distance and increasing stress level were positively associated with the higher likelihood of COVID-19 infection. Additionally, we found that the interaction between stress and wearing masks and washing hands and keeping distance had a significant positive effect on COVID-19 infection. Furthermore, we found that the attributable risk percentage (ARP) for wearing masks, washing hands, and keeping distance was 6.4%% and the ARP for stress was 8.1%, indicating that, if these associations were causa, reducing residents’ stress levels could potentially lead to an 8.1% reduction in COVID-19 infections. Similarly, increasing the frequency of wearing masks, washing hands, and keeping distance could potentially result in a 6.4% decrease in COVID-19 infections.

More than half of the residents have contracted the COVID-19 virus, which has a huge impact on the continued prevention and control of the COVID-19 epidemic. It may be because of the following reasons: The COVID-19 virus mutated into various strains, which is less lethal and pathogenic but spreads faster; in addition, taking into account the epidemic prevention and control situation at that time, Chinese government changed the epidemic prevention and control policy to “Class B epidemic and B Management”, which lifted the social blocking between people; finally, it may be because some areas have higher population densities, which increases the risk of COVID-19 infection. Additionally, the study found that the COVID-19 infection rate in Jiangsu province (67.1%) was significantly higher than that in other three regions, which may be related to population density. The finding is similar to other study [35], in that the higher the population density, the more widespread and rapid the spread of the COVID-19 virus, and the higher the likelihood of contact with susceptible individuals, the higher the likelihood of infection with respiratory viruses, including COVID-19 virus [36, 37], and we found that symptoms of COVID-19 infection in infected people are mostly fever, cough, nasal congestion, runny nose, sore throat, muscle soreness, dizziness, headache, and fatigue and so on.

Residents prior to infection had healthy lifestyle behaviors and emotion. Compared with previous study [38], the lifestyle behaviors and emotion prior to infection of Chinese residents are still very healthy. Besides, women are healthier than men in terms of lifestyle behaviors (smoking, drinking, wearing masks and washing hands and keeping distance), but women are more stressed than men. Gender differences are considered to be an important explanatory parameter for lifestyle behaviors change [39], and women lead healthier lifestyles due to education status, income, self-reliance and the continuous promotion of healthy lifestyle behaviors globally [40, 41], but women are reported to be more affected by stress in all aspects of their lives (such as fertility stress [42], appearance stress [43] and work stress [44, 45], etc.), and their stress is also significantly higher than that of men. Severe psychological and emotional stress will seriously increase the likelihood of COVID-19 infection [11]. Stress among residents with the history of chronic disease and allergic was higher than among residents without chronic diseases, possibly because residents with the history of chronic diseases and allergic were more likely to be infected with the COVID-19 virus [46, 47] and had a higher level of fear of COVID-19 virus [48], resulting in a significant increase in stress among these residents.

Upon adjusting for potential confounding factors, our analysis revealed that there was no significant association between COVID-19 infection and gender. However, we observed a significant association between COVID-19 infection and the educational status of residents. The higher the educational level of the residents, the higher the likelihood of COVID-19 infection. This observation may be attributed to that higher education resources are typically concentrated in urban areas [49]. As a result, residents with higher educational attainment are more likely to reside in urban areas due to occupational opportunities. Urban areas often exhibit high population density, which has been linked to increased susceptibility to COVID-19 infection [35]. Furthermore, our findings indicate that a decreased frequency of wearing masks and practicing proper hand hygiene and physical distancing is associated with an increased likelihood of COVID-19 infection. This correlation can be attributed to the infection mechanism of the COVID-19 virus, which primarily spreads through airborne particles that are inhaled through the respiratory or oral pathways [50, 51]. Wearing masks, practicing hand hygiene, and maintaining physical distance are effective measures in controlling the transmission of the virus that causes COVID-19 [52]. Therefore, a decreased adherence to these preventive measures is associated with a higher risk of COVID-19 infection. Lastly, our study revealed a positive association between residents’ stress levels and the likelihood of COVID-19 infection, which aligns with findings from previous research [11]. Studies have shown that higher stress levels increase the risk of multiple chronic diseases and reduce the self-efficacy of vaccination, the increased incidence of chronic diseases and the reduced self-efficacy of vaccination also increase the risk of COVID-19 infection [26, 47, 53]. Furthermore, the sensitivity analysis confirmed that the estimated effects of the influencing factors on the primary outcomes remained consistent with the original results, further supporting the robustness of our findings.

Finally, we found that the interaction between stress and wearing masks and washing hands and keeping distance had a significant positive effect on COVID-19 infection, and in the correlation analysis, stress was found to be inversely correlated with the frequency of wearing masks and washing hands and keeping distance, suggesting that the higher the stress, the less frequency in wearing masks and washing hands and keeping distance, the higher the likelihood of COVID-19 infection. Our study aligns with previous research that has demonstrated a correlation between psychological factors and lifestyle factors. Specifically, studies have found a positive association between high life risk scores and generalized stress and depression, indicating that higher levels of stress are associated with poorer lifestyle choices [54, 55]. It is possible that residents may experience fear of COVID-19 prior to infection, leading to increased stress levels. This fear may subsequently result in reduced adherence to preventive measures such as wearing masks, washing hands, and maintaining physical distance. The mode of COVID-19 transmission, primarily through airborne particles entering the mouth or respiratory tract, further amplifies the importance of these preventive measures [50, 51]. Therefore, residents can effectively reduce their risk of COVID-19 infection by increasing the frequency of wearing masks, practicing regular hand hygiene, keeping physical distance, and concurrently addressing psychological and emotional stress levels.

Strength and limitation of the study.

This study possesses several notable strengths. Firstly, it stands as the first nationwide large-scale survey conducted in China after the implement of “Class A epidemic and A Management” policy and has enough presentative. Secondly, our study is a current ongoing track study of healthy lifestyle behaviors and emotional conditions, contributing to policymakers’ holistic understanding of Chinese residents’ lifestyle behaviors and emotions. Lastly, we continue to explore the deep association between healthy lifestyle behaviors and emotional expressivity and COVID-19 infection in the new stage, providing new perspectives and new solutions to the complex task of ending the pandemic.

This study is not without its limitations. Firstly, the data relied on self-reported information provided by the participating residents, which introduces the possibility of recall bias or misreporting. Although efforts were made to ensure the accuracy of the data, this inherent limitation should be acknowledged. Secondly, while this study focused on specific lifestyle behavior factors, comprising smoking, drinking, and physical exercise, the exploration of other lifestyle behavior factors and their relationship with COVID-19 infection remains limited. Further research is needed to comprehensively investigate the impact of various lifestyle behavior factors on COVID-19 infection. Future studies can expand the scope to include additional lifestyle behavior variables for a more comprehensive understanding. Thirdly, the findings of this follow-up survey may be limited by the era when COVID-19 infections were declared terminated in most regions of the world. But the evidence presented in this study that healthy lifestyle behaviors and positive emotional expressivity help reduce the risk of COVID-19 infection, provided valuable ideas for future responses to worldwide prevalent unknown emerging infectious diseases.

## Conclusion

In summary, the total COVID-19 infection rate among residents is at a moderate level. It is important for them to continue maintaining these positive aspects of their lifestyle behaviors and emotional well-being. In addition, maintaining preventive measures such as wearing masks, practicing proper hand hygiene, and practicing physical distancing is crucial even when a certain level of balance has been achieved between human resistance and the COVID-19 virus. It is also important for individuals to prioritize their emotional well-being, manage stress levels, and maintain a positive mindset. Being prepared with necessary medications to address symptoms commonly associated with COVID-19, such as fever, cough, and congestion, is also advisable.

### Electronic supplementary material

Below is the link to the electronic supplementary material.

Supplementary Material 1

## Data Availability

The datasets used and/or analyzed during the current study are available from the corresponding author on reasonable request.

## Abbreviations

ACLM: The American College of Lifestyle Medicine;
ARP: Attributable risk percentage;
COVID-19: Coronavirus disease 2019;
DASS-21: Depression, Anxiety and Stress Scale.
