# Supplementary material for: Quantifying the benefits of healthy lifestyle behaviors and emotional expressivity in lowering the risk of COVID-19 infection: a national survey of Chinese population
Source: BMC Public Health. 2023 Nov 30;23:2374. doi: 10.1186/s12889-023-17158-6 (PMC10687789; doi:10.1186/s12889-023-17158-6)
Supplement: Supplementary file 1 — Supplementary Material 1 [file 12889_2023_17158_MOESM1_ESM.docx]

**Additional file1**

**Table S1 Part of the questionnaire**

| **Code:** | | | | |
| --- | --- | --- | --- | --- |
| **Sociodemographic** | | | | |
| 1. Name: | | | | |
| 2. Telephone number: | | | | |
| 3. Age: (years old) | | | | |
| 4. Height: (cm); (kg) | | | | |
| 5. Gender  A. Man B. Woman | | | | |
| 6. Your address: | | | | |
| 7. Marital status  A. Married B. Others | | | | |
| 8. Education status  A. Junior and below B. Senior C. Undergraduate D.Graduate and above | | | | |
| 9. Chronic disease  A. Yes B. No | | | | |
| 10. The history of allergic  A. Yes B. No C. Not clear | | | | |
| **Lifestyle Behaviors** | | | | |
| 1. Do you smoke?  A. Smoke B. Quit C. Never | | | | |
| 2. Do you drink?  A. Drink B. Quit C. Never | | | | |
| 3. How many frequencies in physical exercise do you conduct per week?  A. Never B. <1 time C. 1-2 times D. 3-5 times E. ≥6 times | | | | |
| 4. How often do you wear masks and wash hands and keep distance per week?  A. Never B. Seldom C. Sometimes D. Often E. Always | | | | |
| **DASS-21** | | | | |
| Items | Never | Sometimes | Often | Always |
| 1. I found hard to wind down |  |  |  |  |
| 2. I was aware of dryness of my mouth |  |  |  |  |
| 3. I couldn’t seem to experience any positive feeling at all |  |  |  |  |
| 4. I experienced breathing difficulty (eg, excessively rapid breathing, breathlessness in the absence of physical exertion) |  |  |  |  |
| 5. I found it difficult to work up the initiative to do things |  |  |  |  |
| 6. I tend to over-react to situations |  |  |  |  |
| 7. I experience trembling (eg, in the hands) |  |  |  |  |
| 8. I felt that I was using a lot of nervous energy |  |  |  |  |
| 9. I was worried about situations in which I might panic and make a fool of myself) |  |  |  |  |
| 10. I felt I had nothing to look forward to |  |  |  |  |
| 11. I found myself getting agitated |  |  |  |  |
| 12. I found it difficult to relax |  |  |  |  |
| 13. I felt down-heart and blue |  |  |  |  |
| 14. I was intolerant of anything that kept me from getting on with what I was doing |  |  |  |  |
| 15. I felt I was close to panic |  |  |  |  |
| 16. I was unable to become enthusiastic about anything |  |  |  |  |
| 17. I felt I wasn’t worth much as a person |  |  |  |  |
| 18. I felt that I was rather touchy |  |  |  |  |
| 19. I was aware of the action of my heart in the absence of physical exertion (eg, sense of heart rate increase, heart missing a beat) |  |  |  |  |
| 20. I felt scared without any good reason |  |  |  |  |
| 21. I felt that life was meaningless |  |  |  |  |

**Fig. S1** The population density of every province


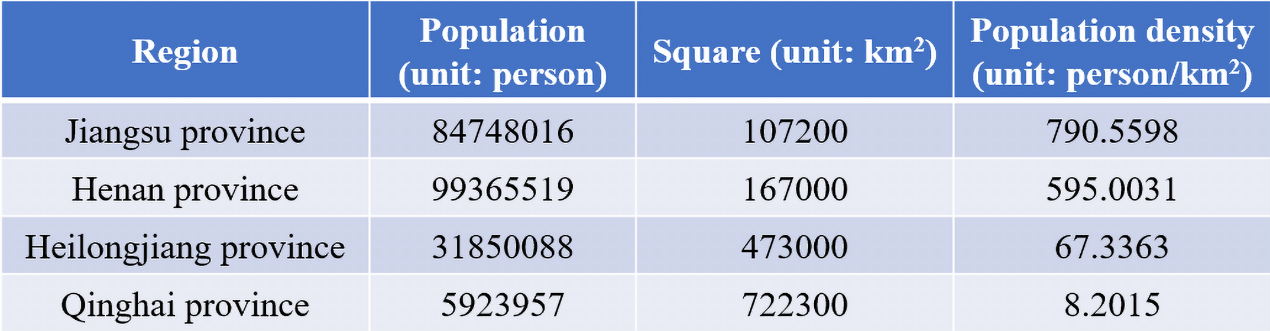


**Table S2** The items of 4 lifestyle behaviors

| 1. Do you smoke?  A. Smoke B. Quit C. Never |
| --- |
| 2. Do you drink?  A. Drink B. Quit C. Never |
| 3. How many frequencies in physical exercise do you conduct per week?  A. Never B. <1 time C. 1-2 times D. 3-5 times E. ≥6 times |
| 4. How often do you wear masks and wash hands and keep distance per week?  A. Never B. Seldom C. Sometimes D. Often E. Always |

**Table S3** The items of DASS-21

| Items | Never | Sometimes | Often | Always |
| --- | --- | --- | --- | --- |
| 1. I found hard to wind down |  |  |  |  |
| 2. I was aware of dryness of my mouth |  |  |  |  |
| 3. I couldn’t seem to experience any positive feeling at all |  |  |  |  |
| 4. I experienced breathing difficulty (eg, excessively rapid breathing, breathlessness in the absence of physical exertion) |  |  |  |  |
| 5. I found it difficult to work up the initiative to do things |  |  |  |  |
| 6. I tend to over-react to situations |  |  |  |  |
| 7. I experience trembling (eg, in the hands) |  |  |  |  |
| 8. I felt that I was using a lot of nervous energy |  |  |  |  |
| 9. I was worried about situations in which I might panic and make a fool of myself) |  |  |  |  |
| 10. I felt I had nothing to look forward to |  |  |  |  |
| 11. I found myself getting agitated |  |  |  |  |
| 12. I found it difficult to relax |  |  |  |  |
| 13. I felt down-heart and blue |  |  |  |  |
| 14. I was intolerant of anything that kept me from getting on with what I was doing |  |  |  |  |
| 15. I felt I was close to panic |  |  |  |  |
| 16. I was unable to become enthusiastic about anything |  |  |  |  |
| 17. I felt I wasn’t worth much as a person |  |  |  |  |
| 18. I felt that I was rather touchy |  |  |  |  |
| 19. I was aware of the action of my heart in the absence of physical exertion (eg, sense of heart rate increase, heart missing a beat) |  |  |  |  |
| 20. I felt scared without any good reason |  |  |  |  |
| 21. I felt that life was meaningless |  |  |  |  |

Note: DASS-21, Depression, Anxiety and Stress Scale 2021.

**Table S4** Factors influencing COVID-19 infection condition of residents (all variables)

| Variables | Model1 | | Model2 | |
| --- | --- | --- | --- | --- |
|  | OR (95%CI) | *p* value | OR (95%CI) | *p* value |
| **Gender** |  |  |  |  |
| Man | Ref. |  |  |  |
| Woman | 1.003 (0.904-1.113) | 0.955 |  |  |
| **Age** |  |  |  |  |
| 18-29 | Ref. |  | Ref. |  |
| 30-39 | 1.064 (0.892-1.270) | 0.490 | 1.318 (1.093-1.590) | 0.004 |
| 40-49 | 1.008 (0.836-1.217) | 0.930 | 1.285 (1.046-1.578) | 0.017 |
| 50-59 | 1.041 (0.865-1.253) | 0.669 | 1.495 (1.206-1.854) | <0.001 |
| ≥60 | 0.829 (0.688-0.999) | 0.049 | 1.186 (0.944-1.490) | 0.143 |
| **Region** |  |  |  |  |
| A province | Ref. |  | Ref. |  |
| B province | 0.447 (0.397-0.504) | <0.001 | 0.523 (0.458-0.596) | <0.001 |
| C province | 0.383 (0.328-0.448) | <0.001 | 0.414 (0.350-0.490) | <0.001 |
| D province | 0.579 (0.440-0.810) | 0.001 | 0.648 (0.474-0.885) | 0.006 |
| **Marital status** |  |  |  |  |
| Married | Ref. |  |  |  |
| Others | 0.859 (0.733-1.007) | 0.061 |  |  |
| **Education status** |  |  |  |  |
| Junior and below | Ref. |  | Ref. |  |
| Senior | 1.149 (1.008-1.309) | 0.037 | 1.130 (0.982-1.300) | 0.088 |
| Undergraduate | 2.051 (1.810-2.325) | <0.001 | 1.943 (1.655-2.282) | <0.001 |
| Graduate and above | 2.111 (1.351-3.300) | 0.001 | 1.674 (1.046-2.678) | 0.032 |
| **Chronic disease** |  |  |  |  |
| Yes | Ref. |  | Ref. |  |
| No | 0.866 (0.752-0.997) | 0.046 | 0.933 (0.794-1.095) | 0.394 |
| **The history of allergic** |  |  |  |  |
| Yes | Ref. |  | Ref. |  |
| No | 0.618 (0.502-0.760) | <0.001 | 0.791 (0.635-0.984) | 0.035 |
| Not clear | 0.603 (0.469-0.775) | <0.001 | 0.763 (0.587-0.992) | 0.044 |
| **Smoking status** |  |  |  |  |
| Yes | Ref. |  |  |  |
| Quit | 1.169 (0.919-1.487) | 0.202 |  |  |
| Never | 1.092 (0.962-1.240) | 0.174 |  |  |
| **Drinking status** |  |  |  |  |
| Yes | Ref. |  |  |  |
| Quit | 0.894 (0.701-1.139) | 0.363 |  |  |
| Never | 0.919 (0.814-1.036) | 0.167 |  |  |
| **Physical exercise** |  |  |  |  |
| Never | Ref. |  | Ref. |  |
| Below 1 time | 1.444 (1.130-1.846) | 0.003 | 1.134 (0.879-1.464) | 0.334 |
| 1-2 times | 1.299 (1.075-1.568) | 0.007 | 1.148 (0.944-1.396) | 0.167 |
| 3-5 times | 1.250 (1.036-1.509) | 0.020 | 1.174 (0.965-1.428) | 0.109 |
| 6 times and above | 1.142 (0.931-1.401) | 0.203 | 1.128 (0.911-1.396) | 0.270 |
| **Wearing masks, washing hands, keeping distance** |  |  |  |  |
| Never | Ref. |  | Ref. |  |
| Seldom | 2.102 (1.431-3.087) | <0.001 | 1.615 (1.087-2.401) | 0.018 |
| Sometimes | 1.878 (1.310-2.693) | 0.001 | 1.456 (1.004-2.112) | 0.047 |
| Often | 1.501 (1.092-2.063) | 0.012 | 1.413 (1.020-1.958) | 0.038 |
| Always | 1.357 (0.988-1.864) | 0.060 | 1.300 (0.937-1.802) | 0.116 |
| **Anxiety** | 1.018 (1.006-1.031) | 0.005 | 0.978 (0.954-1.004) | 0.095 |
| **Depression** | 1.011 (0.999-1.023) | 0.064 |  |  |
| **Stress** | 1.024 (1.014-1.035) | <0.001 | 1.027 (1.005-1.050) | 0.015 |

Note:

Model1, unadjusted;

Model2, adjusted for significantly statistical variables, including age, region, education status, chronic disease, the history of allergic, physical exercise, wearing masks, washing hands, keeping distance, anxiety, stress.

OR, odds ratio; 95%CI, 95% confidence interval.

**Table S5** Correlation analysis of lifestyle behaviors and emotional expression of residents

| **Variables** | | **Emotional expression** | | |
| --- | --- | --- | --- | --- |
|  |  | Anxiety | Depression | Stress |
| **Lifestyle behaviors** | Smoking status | 0.015^a^ | -0.002^a^ | 0.013^a^ |
|  | Drinking status | -0.016^a^ | -0.027^a^ * | -0.028^a^ * |
|  | Physical exercise | -0.095^a^ *** | -0.110^a^ *** | -0.120^a^ *** |
|  | Wearing masks, washing hands, keeping distance | -0.096^a^ *** | -0.092^a^ *** | -0.073^a^ *** |

Note:

^a^ the *β* value;

**p*<0.05, ***p*<0.01, ****p*<0.001

**Table S6** The proportion of symptoms of infection in different lifestyle behaviors and emotional expression of infected people (%)

| Variables | **Smoking status** | | | **Drinking status** | | | **Physical exercise** | | | | | **Wearing masks, washing hands, keeping distance** | | | | | | **Anxiety** | | | **Depress** | | | **Stress** | | |
| --- | --- | --- | --- | --- | --- | --- | --- | --- | --- | --- | --- | --- | --- | --- | --- | --- | --- | --- | --- | --- | --- | --- | --- | --- | --- | --- |
|  | **Yes** | **Quit** | **Never** | **Yes** | **Quit** | **Never** | **Never** | **＜1 time** | **1-2 times** | **3-5 times** | **≥6 times** | **Never** | **Seldom** | **Sometimes** | **Often** | **Always** | **Abnomal** | | **Normal** | **Abnormal** | | **Normal** | **Abnormal** | | **Normal** |  |
| Fever | 74.43 | 70.41 | 76.19 | 75.72 | 69.77 | 75.82 | 73.76 | 79.72 | 75.83 | 76.44 | 71.69 | 70.27 | 76.14 | 76.26 | 77.08 | 73.84 | 82.24 | | 74.7 | 82.35 | | 74.91 | 78.95 | | 75.34 |  |
| Cough | 55.4 | 67.35 | 71.52 | 63.41 | 64.53 | 69.9 | 67.02 | 72.6 | 64.87 | 70.3 | 67.15 | 63.51 | 63.45 | 73.74 | 66.19 | 69.35 | 74.14 | | 67.21 | 76.05 | | 67.25 | 70.18 | | 67.83 |  |
| Nasal congestion, runny nose | 33.94 | 39.29 | 41.26 | 35.14 | 33.72 | 41.79 | 40.43 | 43.77 | 37.96 | 40.12 | 39.2 | 35.14 | 49.24 | 43.88 | 36.61 | 40.56 | 44.86 | | 39.02 | 43.28 | | 39.32 | 41.23 | | 39.55 |  |
| Sore throat | 42.47 | 52.04 | 56.8 | 47.46 | 50 | 56.13 | 52.13 | 57.65 | 52.25 | 55.6 | 50.64 | 52.7 | 56.35 | 56.83 | 51.44 | 54.57 | 54.52 | | 53.41 | 55.46 | | 53.37 | 59.65 | | 53.3 |  |
| Muscle soreness | 49.16 | 45.92 | 49.61 | 48.43 | 52.33 | 49.38 | 46.81 | 47.69 | 52.15 | 50.05 | 44.65 | 43.24 | 52.28 | 51.08 | 47.43 | 50.7 | 54.83 | | 48.66 | 54.62 | | 48.85 | 59.65 | | 49.29 |  |
| Dizziness and headache | 31.05 | 34.69 | 39.31 | 35.75 | 38.37 | 37.82 | 37.59 | 41.64 | 38.45 | 36.71 | 32.85 | 37.84 | 35.53 | 35.61 | 33.51 | 41.8 | 46.11 | | 36.31 | 47.06 | | 36.51 | 50 | | 36.83 |  |
| Arthralgia | 21.46 | 30.1 | 24.11 | 23.67 | 31.4 | 23.44 | 23.4 | 25.62 | 24.95 | 23.37 | 22.5 | 22.98 | 23.86 | 24.46 | 21.03 | 26.86 | 31.78 | | 23.05 | 31.09 | | 23.35 | 31.58 | | 23.65 |  |
| Hyposmia | 15.07 | 16.84 | 20.3 | 16.79 | 20.93 | 19.69 | 19.5 | 17.44 | 19.96 | 19.67 | 16.52 | 24.32 | 14.72 | 15.47 | 17.1 | 22.06 | 24.92 | | 18.33 | 25.21 | | 18.5 | 26.32 | | 18.73 |  |
| Hypogeusia | 19.03 | 22.45 | 26.36 | 21.38 | 26.74 | 25.66 | 23.05 | 23.13 | 26.81 | 23.86 | 23.41 | 31.08 | 18.78 | 17.99 | 23.22 | 27.94 | 31.46 | | 23.82 | 32.35 | | 23.97 | 37.71 | | 24.11 |  |
| Appetite loss | 18.11 | 19.39 | 25.28 | 21.01 | 22.67 | 24.41 | 25.18 | 25.98 | 23.58 | 22.1 | 23.41 | 25.68 | 16.24 | 16.91 | 21.41 | 27.86 | 29.91 | | 22.7 | 30.25 | | 23.56 | 30.7 | | 23.16 |  |
| Diarrhea | 4.57 | 8.67 | 5.63 | 4.71 | 8.14 | 5.73 | 5.67 | 8.54 | 5.19 | 5.84 | 4.36 | 5.41 | 7.61 | 3.96 | 5.37 | 5.88 | 6.23 | | 5.52 | 8.82 | | 5.33 | 7.02 | | 5.54 |  |
| Nausea and vomiting | 1.98 | 4.59 | 7.23 | 3.86 | 4.07 | 6.93 | 6.74 | 4.98 | 6.07 | 6.52 | 4.9 | 6.76 | 5.58 | 4.68 | 5.07 | 7.2 | 7.79 | | 5.77 | 8.4 | | 5.78 | 7.02 | | 5.94 |  |
| Chest tightness and chest pain | 3.96 | 7.65 | 8.14 | 4.59 | 8.72 | 8.14 | 8.51 | 8.54 | 7.73 | 6.82 | 5.81 | 13.51 | 5.58 | 5.04 | 6.96 | 7.89 | 12.46 | | 6.65 | 10.5 | | 6.97 | 13.16 | | 7.02 |  |
| Dyspnea | 2.74 | 5.1 | 5.15 | 4.59 | 3.49 | 4.76 | 6.74 | 4.98 | 5.48 | 3.89 | 3.27 | 2.7 | 0.5 | 3.96 | 3.63 | 6.58 | 10.28 | | 4.01 | 10.92 | | 4.14 | 10.53 | | 4.43 |  |
| Fatigue | 26.48 | 30.1 | 33.38 | 30.68 | 32.56 | 32.09 | 31.91 | 31.67 | 32.58 | 33.59 | 26.68 | 27.03 | 32.99 | 29.5 | 29.73 | 34.37 | 37.69 | | 31.07 | 37.82 | | 31.25 | 38.6 | | 31.49 |  |
| Attention disorder | 4.72 | 4.59 | 5.67 | 5.31 | 7.56 | 5.27 | 8.16 | 7.83 | 4.89 | 5.16 | 4.17 | 4.05 | 4.06 | 3.6 | 4.46 | 7.04 | 10.28 | | 4.86 | 10.92 | | 4.96 | 16.67 | | 0.49 |  |
| Memory loss | 7.46 | 11.22 | 11.26 | 9.9 | 15.7 | 10.26 | 12.77 | 14.23 | 9.78 | 10.13 | 9.26 | 8.11 | 6.09 | 6.47 | 8.93 | 13.7 | 18.69 | | 9.54 | 19.33 | | 9.74 | 26.32 | | 9.87 |  |
| Insomnia | 7.46 | 10.71 | 11.47 | 9.54 | 11.05 | 10.96 | 9.93 | 14.95 | 8.9 | 12.07 | 9.07 | 6.76 | 6.6 | 7.91 | 9.83 | 12.77 | 19.31 | | 9.61 | 18.49 | | 9.95 | 25.44 | | 10.04 |  |
| Weight loss | 4.87 | 7.65 | 7.49 | 6.76 | 8.14 | 6.93 | 7.8 | 8.9 | 7.14 | 7.59 | 3.99 | 6.76 | 3.55 | 6.83 | 6.58 | 7.89 | 9.97 | | 6.62 | 7.56 | | 6.91 | 9.65 | | 6.85 |  |
| Rash | 1.22 | 2.55 | 1.34 | 1.09 | 1.74 | 1.48 | 1.42 | 2.14 | 1.37 | 1.27 | 1.27 | 0 | 1.52 | 0.36 | 1.29 | 1.78 | 3.74 | | 1.13 | 4.62 | | 1.13 | 6.14 | | 1.21 |  |

Note:

Smoke status: 1=yes, 2=quit,3=no;

Drink status: 1=yes, 2=quit,3=no;

Physical exercise: 1=never, 2=below 1 time, 3=1-2 times, 4=3-5 times, 5=6 times and above;

Wearing masks, washing hands, keeping distance: 1=never, 2=seldom, 3=sometimes, 4=often, 5=always;

Anxiety: 1=abnormal, 2=normal;

Depression: 1=abnormal, 2=normal;

Stress: 1=abnormal, 2=normal.

**Table S7** Sensitivity Analysis

| Variables | Model1 | | Model2 | |
| --- | --- | --- | --- | --- |
|  | OR (95%CI) | *p* value | OR (95%CI) | *p* value |
| **Gender** |  |  |  |  |
| Man | Ref. |  |  |  |
| Woman | 0.991 (0.890-1.104) | 0.871 |  |  |
| **Age** |  |  |  |  |
| 18-29 | Ref. |  |  |  |
| 30-39 | 1.097 (0.911-1.321) | 0.327 |  |  |
| 40-49 | 1.010 (0.829-1.230) | 0.922 |  |  |
| 50-59 | 1.044 (0.860-1.267) | 0.662 |  |  |
| ≥60 | 0.872 (0.718-1.060) | 0.168 |  |  |
| **Region** |  |  |  |  |
| A province | Ref. |  | Ref. |  |
| B province | 0.451 (0.398-0.510) | <0.001 | 0.510 (0.447-0.582) | <0.001 |
| C province | 0.380 (0.323-0.447) | <0.001 | 0.410 (0.345-0.487) | <0.001 |
| D province | 0.634 (0.461-0.873) | <0.001 | 0.705 (0.509-0.976) | 0.035 |
| **Marital status** |  |  |  |  |
| Married | Ref. |  |  |  |
| Others | 0.811 (0.685-0.959) | 0.014 | o.638 (0.533-0.764) | <0.001 |
| **Education status** |  |  |  |  |
| Junior and below | Ref. |  | Ref. |  |
| Senior | 1.159 (1.013-1.326) | 0.032 | 1.148 (0.998-1.319) | 0.053 |
| Undergraduate | 2.030 (1.781-2.314) | <0.001 | 1.957 (1.696-2.259) | <0.001 |
| Graduate and above | 2.154 (1.322-3.511) | 0.002 | 1.813 (1.095-3.001) | 0.021 |
| **Chronic disease** |  |  |  |  |
| Yes | Ref. |  | Ref. |  |
| No | 0.892 (0.767-1.036) | 0.134 |  |  |
| **Smoking status** |  |  |  |  |
| Yes | Ref. |  |  |  |
| Quit | 1.117 (0.872-1.431) | 0.381 |  |  |
| Never | 1.061 (0.930-1.210) | 0.377 |  |  |
| **Drinking status** |  |  |  |  |
| Yes | Ref. |  |  |  |
| Quit | 0.887 (0.689-1.141) | 0.350 |  |  |
| Never | 0.910 (0.803-1.031) | 0.137 |  |  |
| **Physical exercise** |  |  |  |  |
| Never | Ref. |  | Ref. |  |
| Below 1 time | 1.425 (1.103-1.841) | 0.007 | 1.123 (0.860-1.465) | 0.393 |
| 1-2 times | 1.303 (1.072-1.585) | 0.008 | 1.148 (0.937-1.405) | 0.183 |
| 3-5 times | 1.238 (1.019-1.504) | 0.031 | 1.169 (0.955-1.430) | 0.129 |
| 6 times and above | 1.170 (0.947-1.445) | 0.145 | 1.145 (0.920-1.426) | 0.226 |
| **Wearing masks, washing hands, keeping distance** |  |  |  |  |
| Never | Ref. |  | Ref. |  |
| Seldom | 1.951 (1.317-2.890) | 0.001 | 1.511 (1.006-2.264) | 0.046 |
| Sometimes | 1.844 (1.276-2.666) | 0.001 | 1.486 (1.016-2.173) | 0.041 |
| Often | 1.454 (1.053-2.007) | 0.023 | 1.406 (1.010-1.957) | 0.044 |
| Always | 1.324 (0.959-1.828) | 0.088 | 1.314 (0.943-1.831) | 0.107 |
| **Anxiety** | 1.015 (1.002-1.029) | 0.025 | 0.982 (0.956-1.009) | 0.191 |
| **Depression** | 1.008 (0.995-1.021) | 0.208 |  |  |
| **Stress** | 1.022 (1.010-1.034) | <0.001 | 1.026 (1.002-1.050) | 0.030 |

Note:

Model1, unadjusted;

Model2, adjusted for significantly statistical variables, including region, marital status, education status, physical exercise, wearing masks, washing hands, keeping distance, anxiety, stress.

OR, odds ratio; 95%CI, 95% confidence interval.
